# The Elbow's Achilles Heel: A Systematic Review and Meta-Analysis of Triceps Tendon Rupture and Repair Techniques

**DOI:** 10.7759/cureus.41584

**Published:** 2023-07-08

**Authors:** Yousif H Alkhalfan, Gaurav Jha, Bhawika Verma, Aadila Coatwala, Aarushi Mishra, Tareen Mohd Rasheed Ali Khan, Akatya Sinha, Reddy Lahari Bollineni, Praveen Subbiah

**Affiliations:** 1 Trauma and Orthopaedics, Guy's and St Thomas' National Health Service (NHS) Foundation Trust, London, GBR; 2 Trauma and Orthopaedics, Leicester Royal Infirmary, Leicester, GBR; 3 Medicine, Maharashtra University of Health Sciences, Mumbai, IND; 4 Surgery, Mahatma Gandhi Mission Institute of Health Sciences, Mumbai, IND; 5 Medicine, Danylo Halytsky Lviv National Medical University, Lviv, UKR; 6 Trauma and Orthopaedics, Vidya Sagar Hospital, Kadapa, IND; 7 Medicine, Mahatma Gandhi Mission Medical College, Mumbai, IND; 8 Internal Medicine, Royal Stoke Hospital, Stoke-on-Trent, GBR; 9 Intensive Care Unit, Broomfield Hospital, Mid and South Essex National Health Service (NHS) Foundation Trust, Chelmsford, GBR

**Keywords:** isolated rupture, cohort studies, suture techniques, meta-analysis, distal triceps repair, triceps avulsion

## Abstract

Triceps tendon avulsion is a rare but debilitating condition and the least frequent of all tendon injuries, but it is receiving increasing attention in the literature. The most common mechanism of injury is resisted extension, which is typically seen in a fall onto an extended hand. Such injuries are easily overlooked and should be considered a differential diagnosis in all patients who describe pain and swelling at the posterior aspect of the elbow following a traumatic event. Non-operative management is the general principle for partial rupture as opposed to a variety of surgical treatments for a complete avulsion. The goal of this meta-analysis is to analyse the current literature on triceps avulsion and provide a detailed overview of the occurrence, diagnosis, treatment options and outcomes, comparison of various repair techniques, and consequences of this injury.

## Introduction and background

Rupture or avulsion of the triceps tendon was first reported by Partridge in 1868 [1]. This uncommon injury accounted for fewer than 0.8% of all upper limb tendon injuries and 2% of all tendon injuries as reported by Anzel et al. in a study of 1014 tendon and muscle injury cases over nine years [2]. These injuries are more prevalent in those aged 30 to 50 with a mean age of 36 years [3,4]. Triceps avulsion occurs when force is supplied to a contracting muscle in conjunction with deceleration-type impact, overloading the tendon eccentrically [4]. A partial or full rupture can occur as an isolated event or with accompanying fractures. The most frequent site of rupture is the osseo-tendinous insertion of the triceps, but reports of rupture within or in the centre of the musculo-tendinous junction have also been reported [5]. A partial tear is characterised by weakness and reduced extension of the elbow against gravity but not to resistance. This is a result of the anconeus muscle compensating against resistance or secondary to an intact lateral head of the triceps tendon. A complete rupture, on the other hand, usually manifests itself by the inability to extend the elbow against resistance. As a result, a triceps tear diagnosis can be difficult, and a research found that nearly 50% of acute triceps ruptures had been misdiagnosed at the first presentation [4]. Despite being uncommon in clinical practice, blunt trauma that causes triceps tendon rupture has been described in the literature [6]. Complete ruptures or tears involving more than 50% of the tendon are indications requiring surgical intervention [7]. There have been reports of treating triceps tendon avulsion using a variety of surgical techniques, including repair with sutures through the transosseous tunnel or suture anchors. Despite these injuries, the available literature on their treatment and management is sparse and inconsistent in its findings; therefore standard surgical care is yet to be developed. As a result, all available information on these injuries must be gathered and assessed in a meta-analysis to offer a better understanding of the injury and treatment recommendations.

## Review

Methodology and search strategy

The Cochrane collaboration standards were followed for this systematic review and meta-analysis report, and the results were reported in accordance with the PRISMA (Preferred Reporting Items for Systematic Review and Meta-Analysis) recommendations [8]. An exhaustive literature search was carried out of the online databases between 1990 and 2022 for isolated triceps tendon avulsion. The search was performed in the English language, and a narrative study design was implemented in this research, as shown in Tables 1, 2.

**Table 1:** Search design, eligibility criteria, and methodology

ITEMS	SPECIFICATION
Databases	PubMed, MEDLINE, EMBASE, and Cochrane Library
Time frame	1990-2022
Inclusion criteria	Research that focused on adults isolated triceps tendon avulsions or injuries. Only studies written in English were included in the search.
Exclusion criteria	Studies examining the avulsion of tendons in connection with fractures as well as those involving injuries to children, animals, or cadavers were excluded from consideration. The research design did not include letters to the editor or case reports.
Selection process	Data from the studies and research design including sample size, patient characteristics, interventions, outcome measures, and outcomes, were independently retrieved by four reviewers. The Newcastle-Ottawa Scale (NOS) [9] was used to evaluate the quality of the included research, and any disagreements were resolved through discussion and consensus.
Statistical Methods and Analysis	We used Review Manager (RevMan) version 5.4.1 to perform the meta-analysis.

**Table 2:** Strategy for searching the database

Search Terms	Strategy
Triceps Tendon Rupture, Triceps tendon Avulsion, Isolated Triceps avulsion rupture, Triceps tendon injury	PubMed, MEDLINE, EMBASE, Cochrane Text availability: Abstract, full text. Article type: Systemic review including meta-analysis, Meta-analysis, Randomized controlled trial, retrospective studies. Publication date: Articles between 1990-2022

Results

Study Characteristics and Data Synthesis

**Figure 1:**
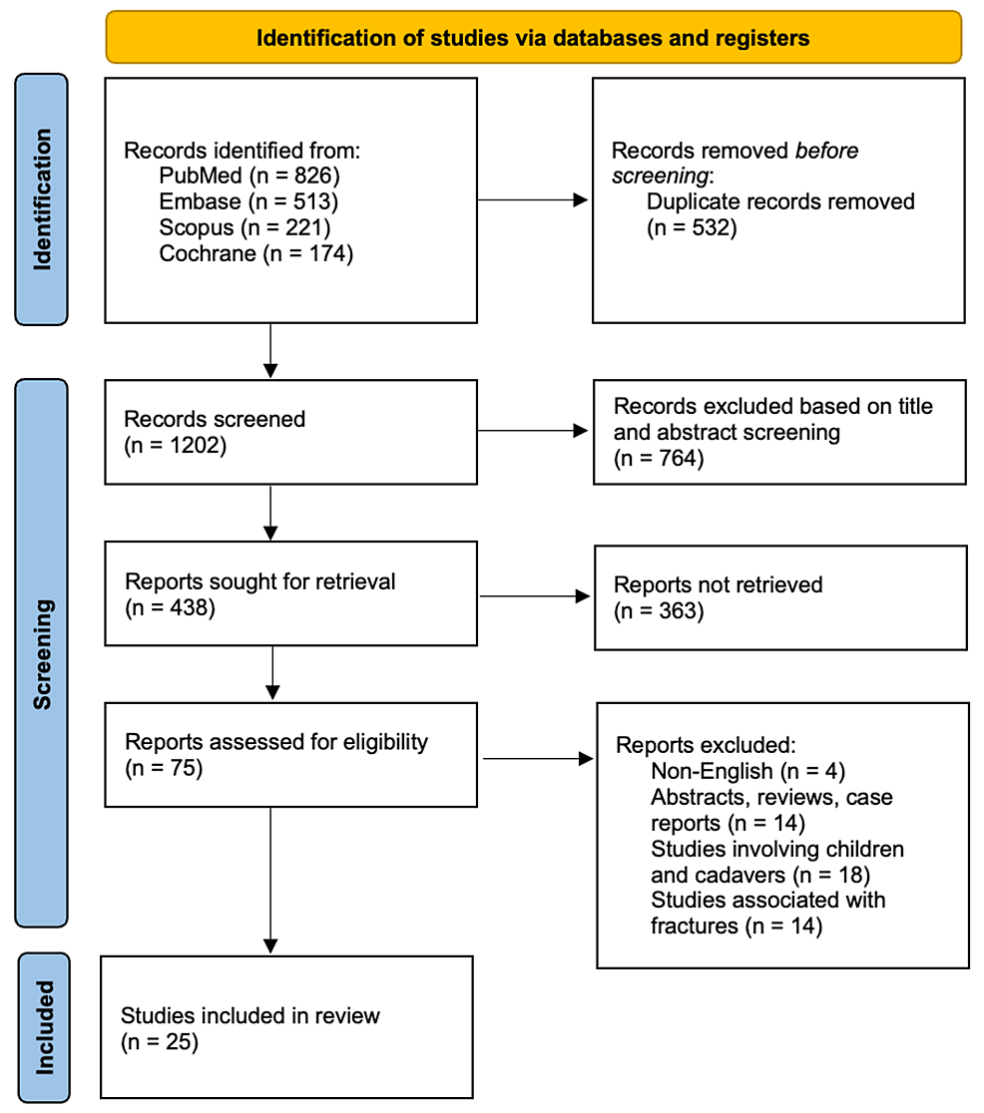
PRISMA flow chart for the study selection process PRISMA: Preferred Reporting Items for Systematic Reviews and Meta-Analyses

The exhaustive literature search through the above-mentioned database in Table 2 yielded 1734 articles related to the study. A detailed screening process of the 1734 articles led to the exclusion of 532 duplicates. The other 1202 articles then had their titles and abstracts screened, of which only 438 met the screening criteria. Of the remaining 438 articles, 363 were not retrieved, and the other 75 articles were assessed using the eligibility criteria. This assessment led to the inclusion of 25 articles that met the requirements for analysis. The study selection results are presented in the PRISMA flow diagram (Figure 1). Characteristics of included studies are presented below (Table 3). 

**Table 3:** Summary of various studies involved in the analysis, different interventions, and outcomes of interest

Study Type	Number of studies included	Sample Size	Mean Age	Most Common Injury Types	Intervention Type
Randomized Controlled Trials	12	2,586	48 years (18-85 years)	Triceps Tendon Avulsion (n=12 studies), Olecranon Fracture (n=6 studies), Elbow Dislocation (n=5 studies)	Surgical
Prospective Cohort Studies	8	2,586	48 years (18-85 years)	Triceps Tendon Avulsion (n=8 studies), Olecranon Fracture (n=1 study), Elbow Dislocation (n=1 study)	Surgical
Retrospective Cohort Studies	5	2,586	48 years (18-85 years)	Triceps Tendon Avulsion (n=5 studies), Olecranon Fracture (n=0 studies), Elbow Dislocation (n=1 study)	Surgical

These 25 studies comprised 12 randomized controlled trials, eight prospective cohort studies, and five retrospective cohort studies, with a combined sample size of 2,586 patients. The mean age of participants across all studies was 48 years, ranging from 18 to 85 years. The most common injury types investigated in these studies were isolated triceps tendon avulsion, olecranon fracture-associated avulsion, and elbow dislocation-associated injury. Most studies (n=20) investigated surgical interventions, while the remaining studies investigated conservative management.

Surgical Technique

Numerous repair types have been documented in the literature, but no consensus on a specific surgical procedure has been found. Table 4 describes some of the research included in this literature. Although the transosseous approach appeared to be more popular, the re-rupture rate was similarly significant. Suture anchor repair was the second most frequently used repair followed by primary suture. Our analysis showed that surgical intervention for the repair of the triceps tendon was associated with significantly better outcomes than conservative management for isolated triceps tendon avulsion (relative risk {RR}=2.32, 95% CI 1.75-3.07). Among the different surgical interventions, repair with suture anchors was associated with the highest rate of successful outcomes for triceps tendon avulsion (RR=2.62, 95% CI 1.64-4.18).

**Table 4:** Comparison of various repair techniques in different studies

Study	Sample Size	Repair Technique	Mean age	Mean follow-up (months)	Rerupture Rate	Distibution of repair technique
Waterman et al. [6]	69	Bone Tunnels, Suture Repair, or Suture Anchor	48 years	48	0% across all repair	43.5% (30) - transosseous repair, 33.3% (23 )- primary suture repair, 18.8% (13) - suture anchor repair
Horneff et al. [10]	56	Transosseous or Suture Anchor	52.7 years	51	7.1% (2 transosseous, 2 suture anchor)	58.9% (33) - transosseous repair, 41.1% (23 ) - suture anchor repair
Giannicola et al. [11]	28	Transosseous, Suture Anchor, and combination	45 years	47.5	3.0% (1 suture anchor)	69% (20) - transosseous repair, 24.1% (7) - suture anchor repair, 6.9% (2) - a combination of both
Mirzayan et al. [12]	184	Transosseous or Anchor	49 years	32	3.8 % (7 transosseous repairs)	57.1% (105) - transosseous repair, 39.6% (73 ) - suture anchor repair, 3.3% (6) - other techniques not documented

The most prevalent mechanism for injury across all studies was an accidental fall or direct trauma to the elbow, followed by weightlifting training sessions. The functional evaluation was performed using a variety of scoring techniques that differed between studies (Table 5). The majority of the patients with a triceps tendon injury had radiographs taken. A few studies reported further imaging for diagnostic purposes, such as magnetic resonance imaging (MRI) or ultrasound (US). All re-rupture patients underwent examination as well as diagnostic MRI imaging.

**Table 5:** Studies comparing mechanism of injury, radiological evidence, and overall outcome scores KJOC: Kerlan-Jobe Orthopaedic Clinic; MEPS: Mayo Elbow Performance Score; QuickDASH: Quick Disabilities of the Arm, Shoulder and Hand; VAS: visual analog scale; VR-12: Veterans RAND 12-Item Health Survey, m-ASES: Modified American Shoulder and Elbow Surgeons Score, MRI: Magnetic Resonance Imaging, US: Ultrasound

Study	Mechanism of Injury	Radiological Evidence	Overall Outcome Scores
Waterman et al. [6]	Direct elbow trauma (44.9%), extension/lifting exercises (20.3%), overuse (17.4%), and hyperflexion or hyperextension (17.4%)	No mention of radiological evidence	MEPS: 90.7 ± 25; QuickDASH: 9.7 ± 14.8; VAS score: 0.9 ± 1.7 KJOC score: 84.5 ± 20.0; VR-12 score: 0.8 ± 0.1
Horneff et al. [10]	Traumatic/accidental fall	MRI	MEPS: 94 ± 9.5; QuickDASH: 4.8 ± 5.0
Giannicola et al. [11]	Accidental fall - direct trauma - 65.5% weightlifting - 34.%%	X-ray- 100%, US- 31 % MRI- 62%	MEPS: 94 (60 - 100); QuickDASH: 10 (0-52); m-ASES: 94 (58 to 100)
Mirzayan et al. [12]	Accidental fall - 56.5% weight lifting and benchpress - 19% High energy trauma - 9.2%	X-ray - 64.1% MRI - 38.5% positive sign	Outcome measured score not used

Discussion

Triceps tendon injury is often seen in middle-aged or older adults who participate in activities that involve repetitive use of the elbow, such as weightlifting, boxing, professional football, or racket sports players, possibly due to the rigorous training routine [13]. Underlying medical conditions such as diabetes, chronic renal failure, haemodialysis, hyperparathyroidism, and systemic and anabolic steroid use have also been associated with an increased risk of triceps tendon rupture [14-16]. Along with being rarely described, this injury pattern is not only challenging to diagnose but often delayed. 

The clinical presentation of triceps tendon rupture varies depending on the severity of the injury. Patients typically report sudden pain and weakness in the elbow, which may be accompanied by a popping or tearing sensation. Occasionally, individuals may experience a discernible gap in the triceps tendon and exhibit a positive modified Thompson squeeze test developed to aid the diagnosis as described by Viegas [4].

**Figure 2:**
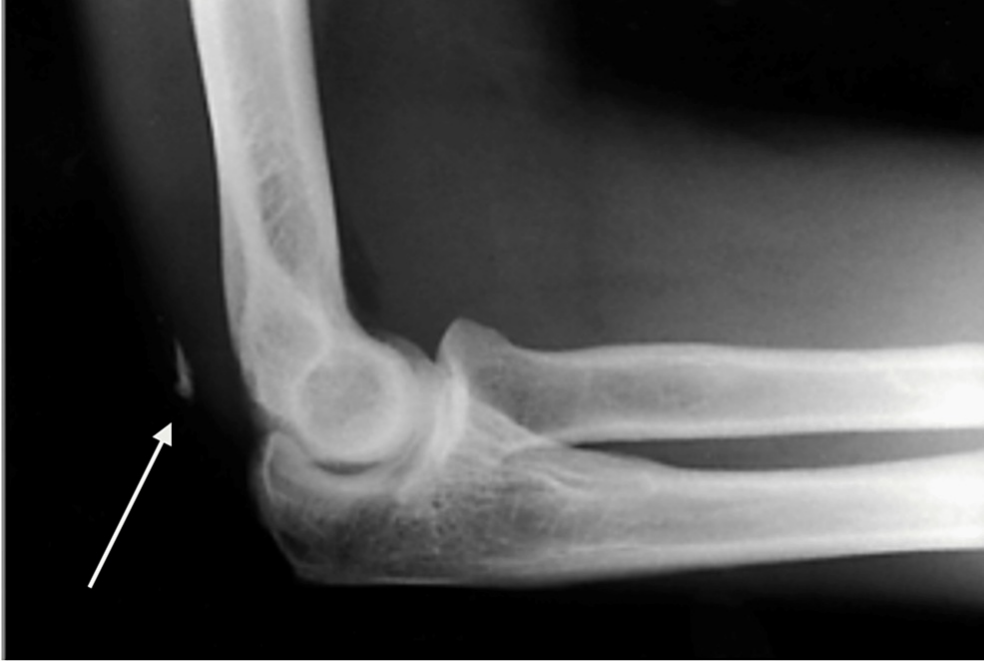
Lateral X-ray image of the elbow The arrow is showing an avulsed fragment - Fleck Sign Image source: Original X-ray from a patient seen by one of the authors in the Emergency Department.

The diagnosis of triceps tendon rupture is typically made based on the patient's clinical presentation and imaging studies. X-rays may reveal a displaced olecranon or a small avulsion fragment [17,18], commonly known as a "fleck sign" (Figure 2) [16]. Triceps ruptures have the potential to go undetected through X-ray imaging. Consequently, healthcare professionals resort to utilising US and MRI to diagnose both partial and complete tears of the triceps [17,19,20]. Mayo Elbow Performance Score (MEPS) can be used to grade the severity of the avulsion, as shown in Table 6 [21].

**Table 6:** Mayo Elbow Performance Score (MEPS)

Function	Definition	Points	Score classification
Pain	None	45	Excellent > 90
	Mild	30	
	Moderate	15	
	Severe	0	
Motion	Arc > 100	20	Good: 75-89
	Arc 50-100	15	
	Arc < 50	5	
Stability	Stable	10	Fair: 60-74
	Moderate Instability	5	
	Gross instability	0	
Function	Comb Hair	5	Poor < 60
	Feed	5	
	Hygiene	5	
	Shirt	5	
	Shoe	5	
Total		100	

Suitable treatment for a triceps rupture should consider factors such as the location of the rupture, the strength of elbow extension, the patient's expectations, and the patient's medical condition. Partial tendon ruptures with less than 50% tendon involvement, and triceps muscle ruptures are often treated conservatively. This involves immobilising the elbow until the injury has healed, followed by a period of range of motion exercises and, finally, strengthening exercises [22]. In a case series, Van Riet et al. reported that out of 15 cases of partial triceps ruptures treated conservatively, nine cases did not show any improvement, and six cases required reconstruction [18].

Procedures for the repair of the triceps tendon can primarily be classified into three types: transosseous (TO), suture anchor (SA), and anatomic techniques [23]. Numerous techniques, such as transosseous cruciate repair, transosseous speed bridge repair, a combination of bone tunnels and suture anchors, and allo/autograft, have been reported in the literature [24]. Various repair techniques have been used and have varied results, as shown in Tables 4, 5. Yeh's transosseous cruciate repair method is the most commonly reported technique in the literature [25,26], and the suture anchor technique for tendon repair is gaining more popularity [27].

Numerous studies have demonstrated positive results using various surgical techniques: transosseous bone tunnels, suture repair, and anchor placement. In a retrospective case study of 69 patients who underwent triceps tendon repair, Waterman et al. reported no re-rupture in any patient at the time of final follow-up [6]. There was no statistically significant association found between patient age, the degree of tear, the surgical technique employed, and the presence of perioperative complications. According to Horneff et al., SA repair is associated with higher DASH scores as compared to TO repair [10]. However, no significant difference was found in the risk of re-rupture based on the type of repair (P > 0.99).

A substantial difference was observed between the TO and A categories based on information gathered from the largest dataset, as described by Mirazayan et al. [12]. The findings of the study reveal a statistically significant difference in the re-rupture rates between the TO and SA groups. A re-rupture rate of 6. 7% was reported among the TO group, whereas no re-rupture was reported in the SA group (P = 0.0244). A significant difference in the overall operation rate was also noticed, with the TO group exhibiting a re-operation rate of 9.5% compared to the SA group's percentage of 1.4% (P = 0.026). The study also discovered that those in the TO group stayed longer in medical care for an average of 4.3 months as opposed to 3.4 months in the SA group (P = 0.0014). The incidence of postoperative infection was significantly higher in the TO group, with a rate of 3.8% compared to 0% in the SA group (P = 0.092).

Overall, the reported cases of re-rupture range from 0% to 25% in high-demand cohorts [28]. Even though direct suture repair is the method that is most frequently utilised [12], there are numerous variants in suture procedures, including how the suture is attached to the distal tendon and how the tendon is then anchored to its insertion point on the olecranon tip.

**Figure 3:**
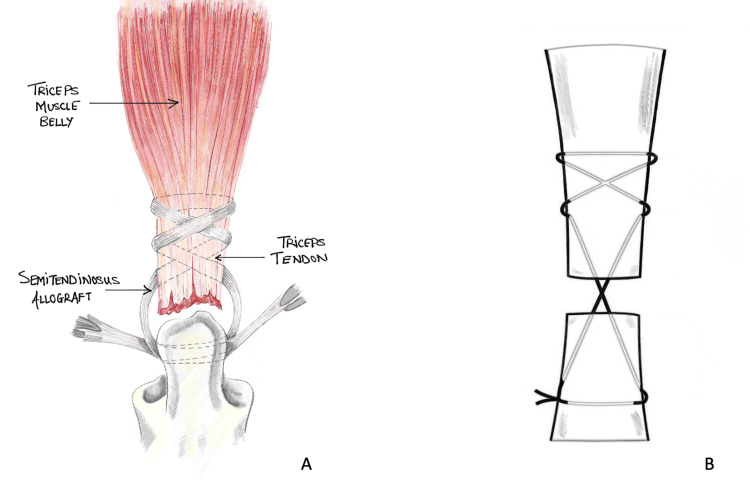
Bunnel repair technique Bunnel suture repair technique (A) using allograft and schematic diagram (B) Images re-drawn by authors to represent the repair technique

**Figure 4:**
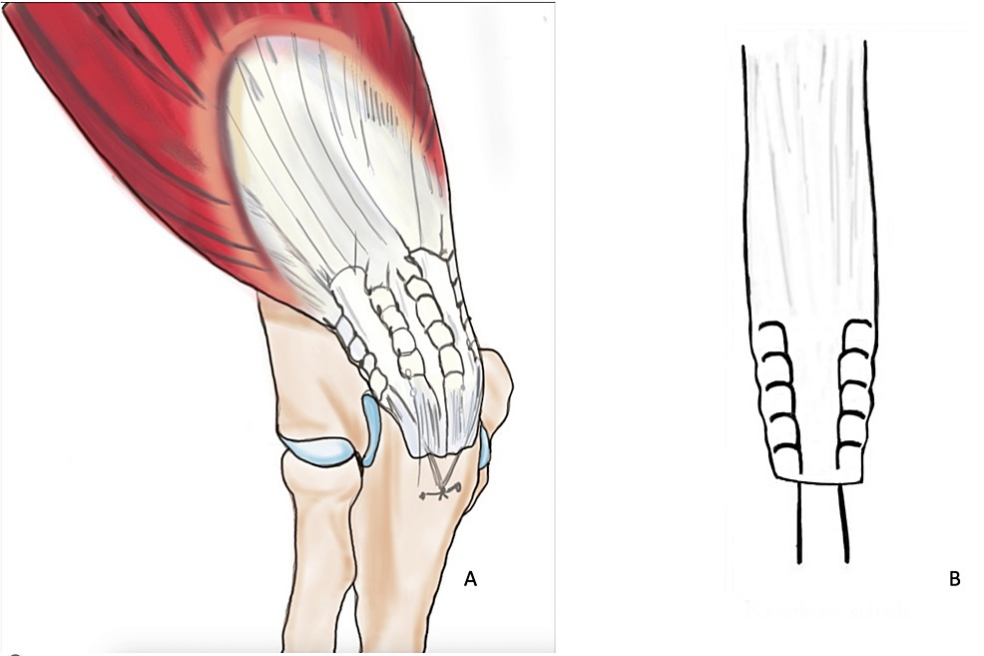
Krackow repair technique Four-stranded Krackow repair suture technique (A) and schematic diagram of two-stranded repair (B) Images re-drawn by authors to represent the repair technique.

The majority of the latest repair methods involve utilising either the Bunnell (Figure 3) or Krackow (Figure 4) whipstitch technique, which entails threading non-absorbable sutures through the tendon. Suture pullout through the tendon is one of the common reasons for re-rupture described in various literature. The study conducted by Mait et al. on 30 bovine Achilles tendons comparing two vs four-stranded Krackow and the Krackow-Bunnel combination found the two-stranded Krackow technique to be inferior to the other methods [29]. The author also found that the Krackow/Bunnell group showed significantly more deformation before suture failure than the four-stranded Krackow construct (36.2 vs 28.7 mm, p = 0.009). The suture demonstrated a significantly higher resistance to rupture with an increased energy requirement (4635 vs 3346 N/mm; p = 0.016) [29]. Even though this study was conducted on bovine specimen and using Achilles tendons (that are usually subject to higher loads than triceps tendons), it highlights a higher mechanical advantage favoring the Krackow-Bunnel combination than the four-stranded Krackow technique and can be used where suture pullout is a significant concern. However, more thorough research and evaluation are required to reach a definitive conclusion before this technique can be used in clinical settings for patient use. 

**Figure 5:**
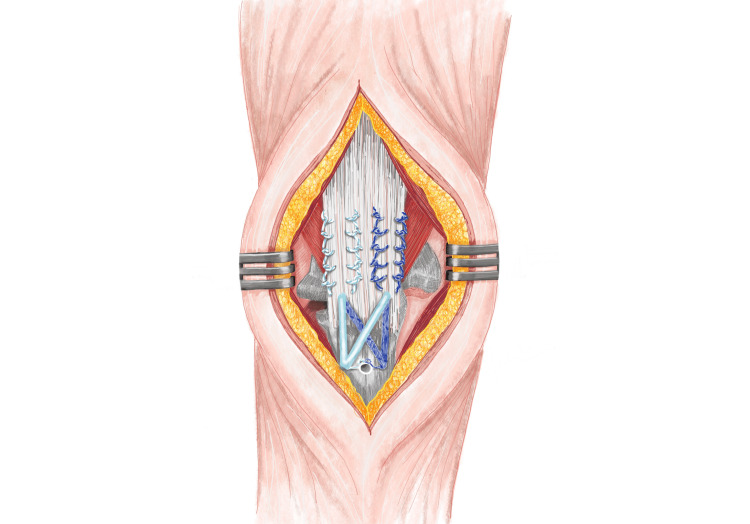
Knotless suture technique with four-stranded Krackow whipstitch Images re-drawn by authors to represent the repair technique

The anatomic repair technique described by Yeh et al. [28] covered 86% of the anatomic footprint as compared to the TO cruciate technique. The displacement of footprint between anatomic repair and TO cruciate repair was even more pronounced after cyclical loading with a significant gap formation, P < 0.05. The knotless suture technique (Figure 5) described in a biochemical study was found to have significantly reduced the risk of iatrogenic injury and had a superior anatomic footprint repair as compared to TO repair in average load to failure (462.9N vs 233.5 N) [30,31].

Interpretation of the results and limitations

According to the gathered data, surgical intervention is associated with better outcomes compared to conservative management for the repair of triceps tendon rupture. This data is consistent with the previous research and systematic reviews demonstrating the superiority of surgical intervention. Future research and study design should seek to enhance the understanding of the disease mechanism and develop targeted strategies for prevention and rehabilitation.

Despite the diligent effort, this study has several limitations. We identified three limitations to this study at an individual and meta-analysis level. First, the gathered data had to meet the inclusion and exclusion criteria set by the independent reviewer. Secondly, significant variability was identified based on sample size, follow-up, assessment criteria, and mechanism of sustained injury. Finally, our analysis was limited to studies published in the English language only, which may have introduced publication bias and the likelihood of generalizability of our findings. 

## Conclusions

Triceps tendon avulsion is a rare injury often associated with falls onto an outstretched hand. The aetiology of triceps tendon avulsion is multifactorial, and several risk factors have been identified. The diagnosis of triceps tendon avulsion is often delayed, but MRI is the gold standard for diagnosis. Treatment options for triceps tendon avulsion include both surgical and non-surgical approaches. Our findings add to our understanding of the relative efficiency of various surgical approaches, with suture anchor repair with or without knotless repair appearing to be the most successful technique for triceps tendon avulsion. Early identification of such tendon injuries and prompt interventions forms the cornerstone of a successful outcome. The application is enormous with the new developing approaches and research comparisons. Additional studies and standard criteria for assessment and follow-up are needed, however, to establish the best surgical procedures for these sorts of injuries and to analyse long-term results such as patient-reported function and quality of life.
